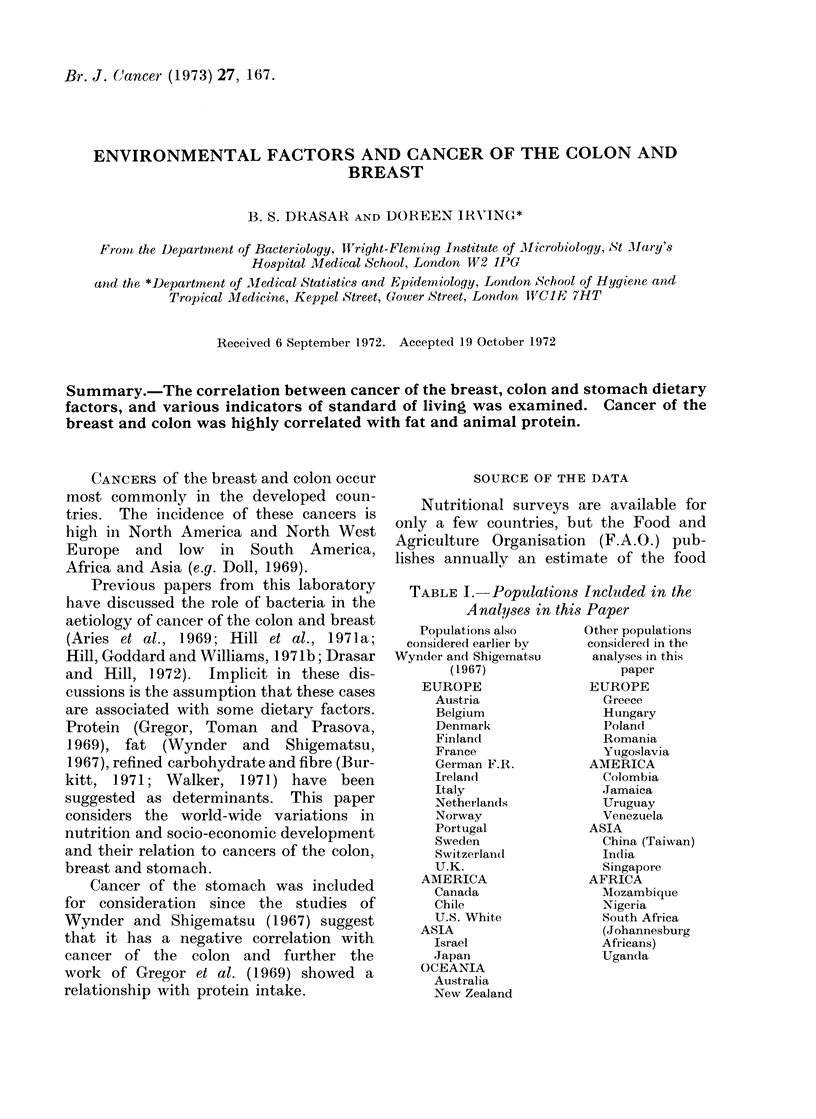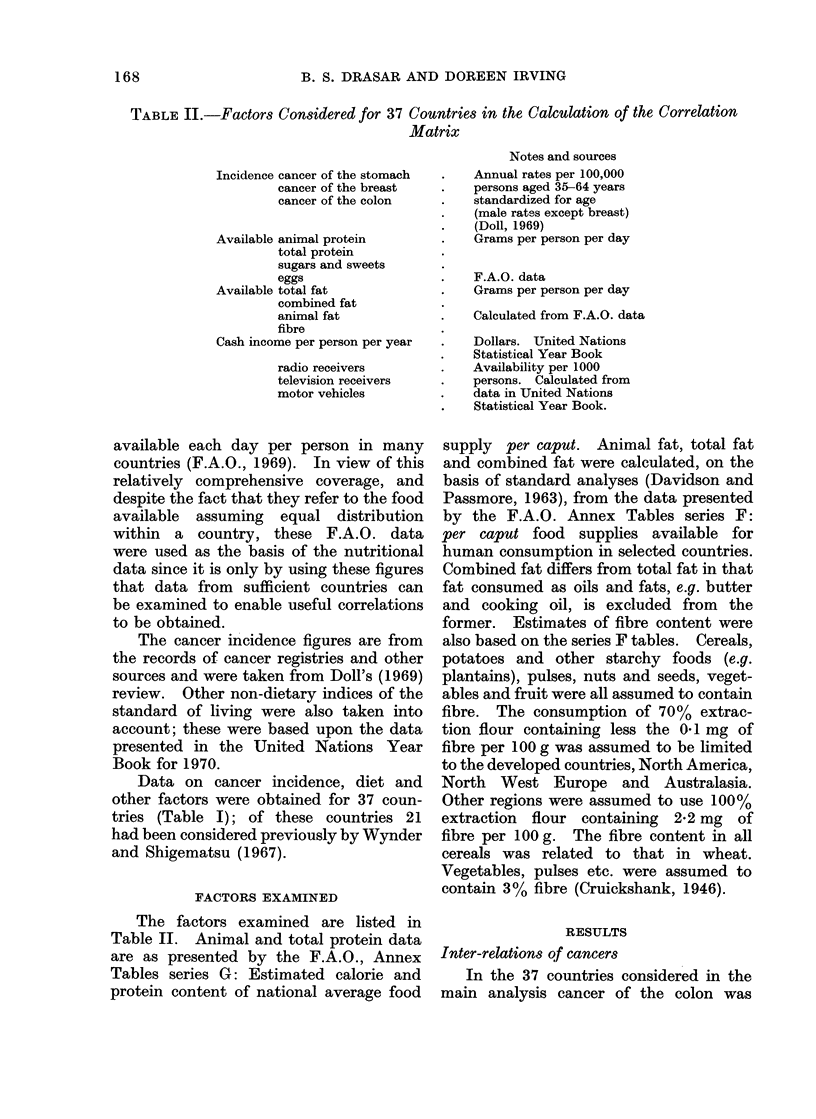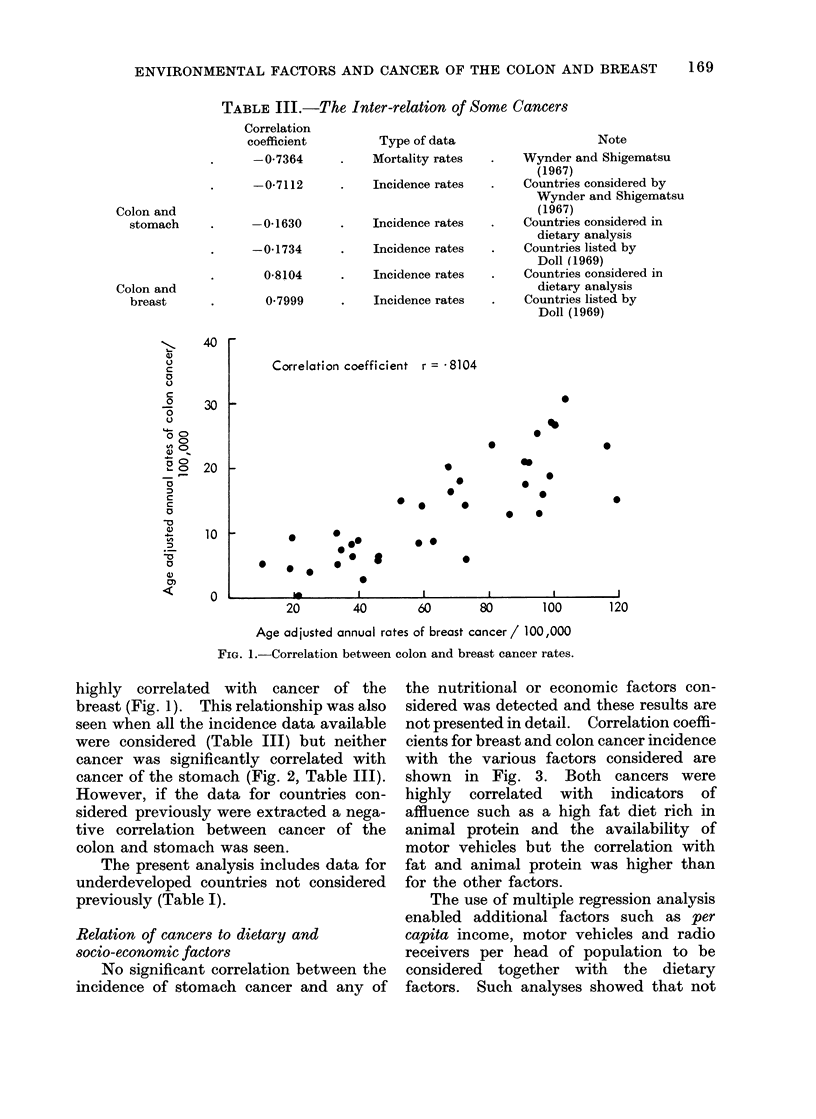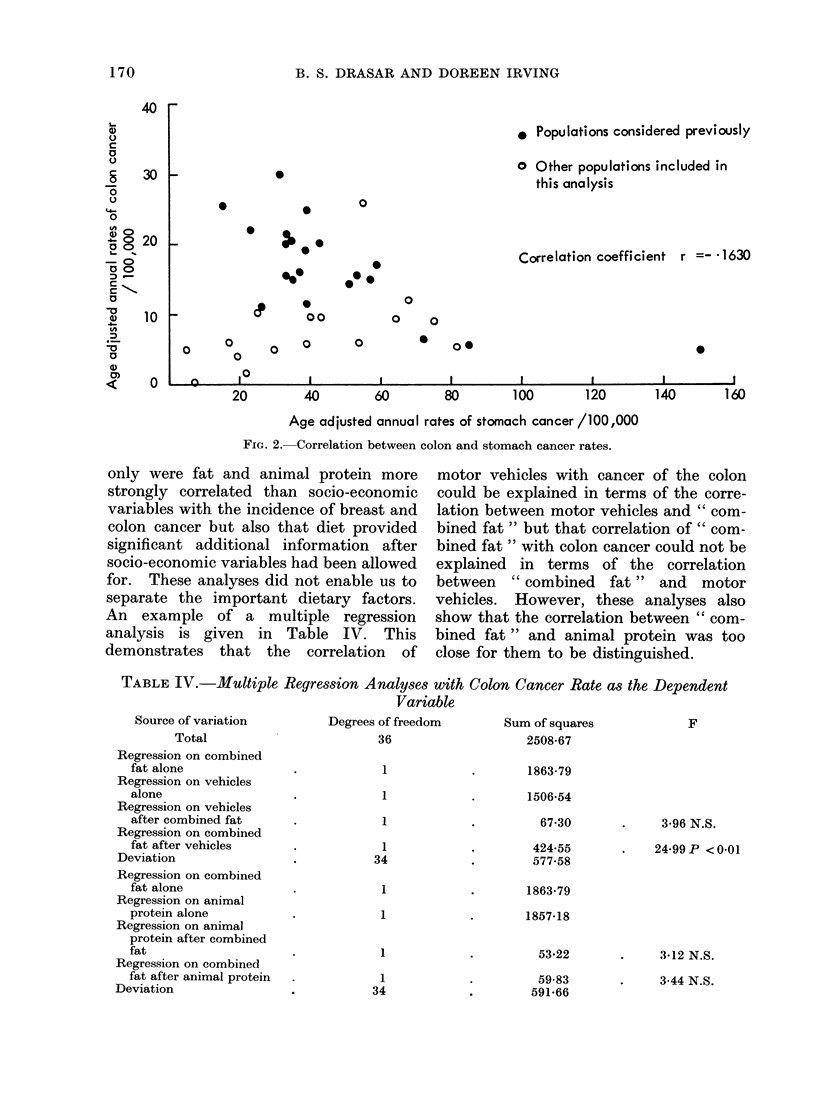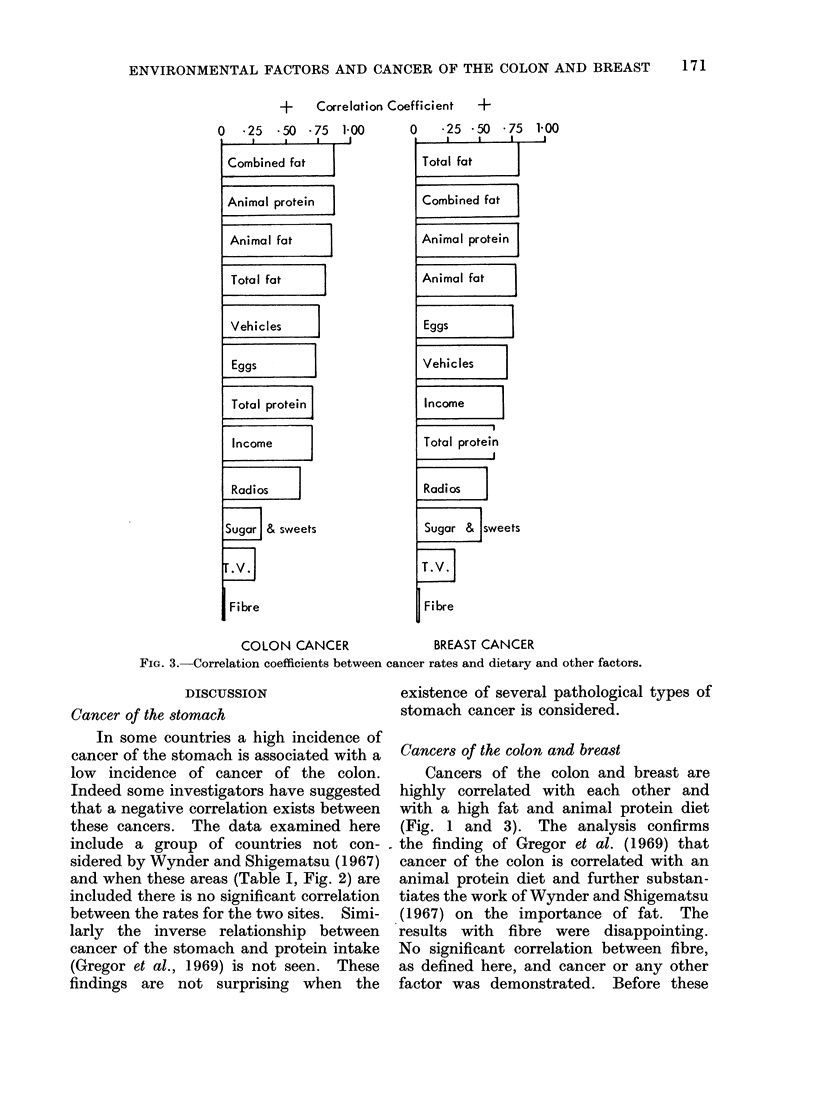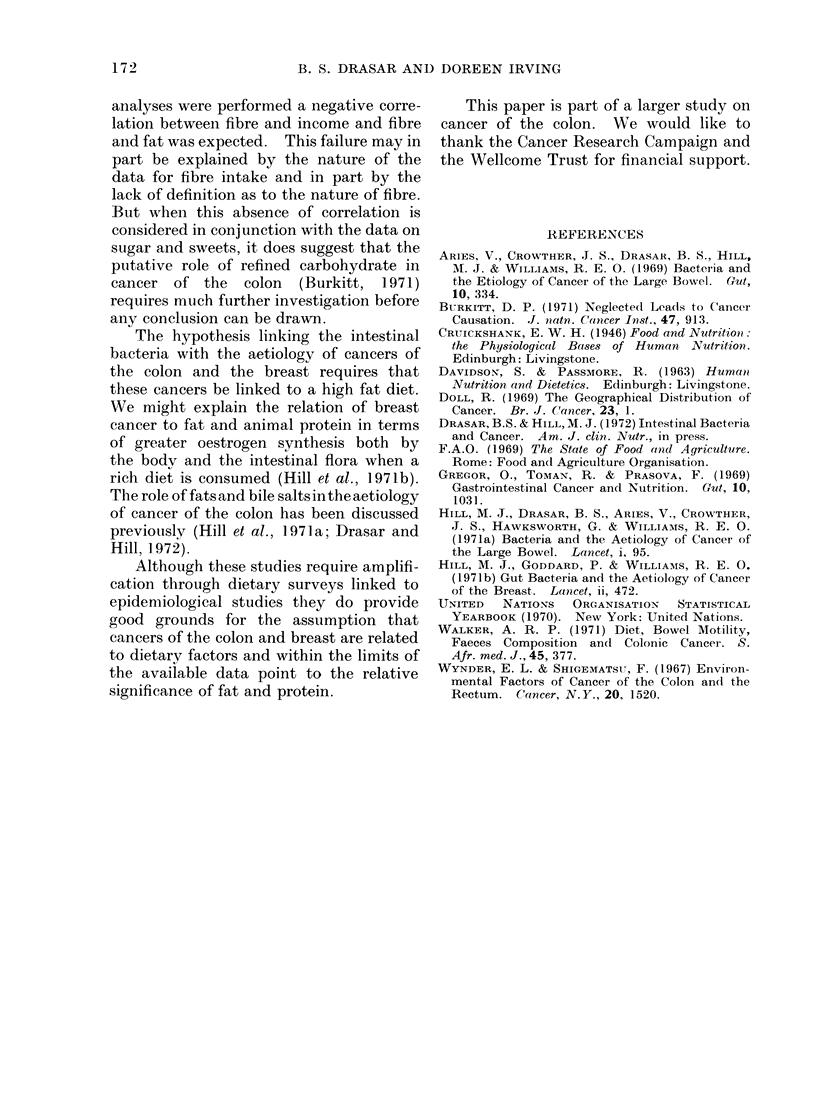# Environmental Factors and Cancer of the Colon and Breast

**DOI:** 10.1038/bjc.1973.20

**Published:** 1973-02

**Authors:** B. S. Drasar, Doreen Irving

## Abstract

The correlation between cancer of the breast, colon and stomach dietary factors, and various indicators of standard of living was examined. Cancer of the breast and colon was highly correlated with fat and animal protein.


*Br. J. Cancer* (1973) **27**, 167.

### ENVIRONMENTAL FACTORS AND CANCER OF THE COLON AND BREAST

B. S. DRASAR AND DOREEN IRVING*

*From the Department of Bacteriology, Wright-Fleming Institute of Microbiology, St Mary's Hospital Medical School, London W2 1PG*

*and the *Department of Medical Statistics and Epidemiology, London School of Hygiene and Tropical Medicine, Keppel Street, Gower Street, London WC1E 7HT*

Received 6 September 1972. Accepted 19 October 1972

**Summary.—The correlation between cancer of the breast, colon and stomach dietary factors, and various indicators of standard of living was examined. Cancer of the breast and colon was highly correlated with fat and animal protein.**

CANCERS of the breast and colon occur most commonly in the developed countries. The incidence of these cancers is high in North America and North West Europe and low in South America, Africa and Asia (*e.g.* Doll, 1969).

Previous papers from this laboratory have discussed the role of bacteria in the aetiology of cancer of the colon and breast (Aries *et al.*, 1969; Hill *et al.*, 1971a; Hill, Goddard and Williams, 1971b; Drasar and Hill, 1972). Implicit in these discussions is the assumption that these cases are associated with some dietary factors. Protein (Gregor, Toman and Prasova, 1969), fat (Wynder and Shigematsu, 1967), refined carbohydrate and fibre (Burkitt, 1971; Walker, 1971) have been suggested as determinants. This paper considers the world-wide variations in nutrition and socio-economic development and their relation to cancers of the colon, breast and stomach.

Cancer of the stomach was included for consideration since the studies of Wynder and Shigematsu (1967) suggest that it has a negative correlation with cancer of the colon and further the work of Gregor *et al.* (1969) showed a relationship with protein intake.

### SOURCE OF THE DATA

Nutritional surveys are available for only a few countries, but the Food and Agriculture Organisation (F.A.O.) publishes annually an estimate of the food

TABLE I.—*Populations Included in the Analyses in this Paper*

| Populations also considered earlier by Wynder and Shigematsu (1967) | Other populations considered in the analyses in this paper |
|---|---|
| EUROPE | EUROPE |
| Austria | Greece |
| Belgium | Hungary |
| Denmark | Poland |
| Finland | Romania |
| France | Yugoslavia |
| German F.R. | AMERICA |
| Ireland | Colombia |
| Italy | Jamaica |
| Netherlands | Uruguay |
| Norway | Venezuela |
| Portugal | ASIA |
| Sweden | China (Taiwan) |
| Switzerland | India |
| U.K. | Singapore |
| AMERICA | AFRICA |
| Canada | Mozambique |
| Chile | Nigeria |
| U.S. White | South Africa |
| ASIA | (Johannesburg |
| Israel | Africans) |
| Japan | Uganda |
| OCEANIA | |
| Australia | |
| New Zealand | |

TABLE II.—*Factors Considered for 37 Countries in the Calculation of the Correlation Matrix*

| | | Notes and sources |
|---|---|---|
| Incidence | cancer of the stomach | Annual rates per 100,000 |
| | cancer of the breast | persons aged 35–64 years |
| | cancer of the colon | standardized for age |
| | | (male rates except breast) |
| | | (Doll, 1969) |
| Available | animal protein | Grams per person per day |
| | total protein | |
| | sugars and sweets | |
| | eggs | F.A.O. data |
| Available | total fat | Grams per person per day |
| | combined fat | |
| | animal fat | Calculated from F.A.O. data |
| | fibre | |
| Cash income per person per year | | Dollars. United Nations |
| | | Statistical Year Book |
| | radio receivers | Availability per 1000 |
| | television receivers | persons. Calculated from |
| | motor vehicles | data in United Nations |
| | | Statistical Year Book. |

available each day per person in many countries (F.A.O., 1969). In view of this relatively comprehensive coverage, and despite the fact that they refer to the food available assuming equal distribution within a country, these F.A.O. data were used as the basis of the nutritional data since it is only by using these figures that data from sufficient countries can be examined to enable useful correlations to be obtained.

The cancer incidence figures are from the records of cancer registries and other sources and were taken from Doll's (1969) review. Other non-dietary indices of the standard of living were also taken into account; these were based upon the data presented in the United Nations Year Book for 1970.

Data on cancer incidence, diet and other factors were obtained for 37 countries (Table I); of these countries 21 had been considered previously by Wynder and Shigematsu (1967).

### FACTORS EXAMINED

The factors examined are listed in Table II. Animal and total protein data are as presented by the F.A.O., Annex Tables series G: Estimated calorie and protein content of national average food supply *per caput*. Animal fat, total fat and combined fat were calculated, on the basis of standard analyses (Davidson and Passmore, 1963), from the data presented by the F.A.O. Annex Tables series F: *per caput* food supplies available for human consumption in selected countries. Combined fat differs from total fat in that fat consumed as oils and fats, *e.g.* butter and cooking oil, is excluded from the former. Estimates of fibre content were also based on the series F tables. Cereals, potatoes and other starchy foods (*e.g.* plantains), pulses, nuts and seeds, vegetables and fruit were all assumed to contain fibre. The consumption of 70% extraction flour containing less the 0·1 mg of fibre per 100 g was assumed to be limited to the developed countries, North America, North West Europe and Australasia. Other regions were assumed to use 100% extraction flour containing 2·2 mg of fibre per 100 g. The fibre content in all cereals was related to that in wheat. Vegetables, pulses etc. were assumed to contain 3% fibre (Cruickshank, 1946).

### RESULTS

### *Inter-relations of cancers*

In the 37 countries considered in the main analysis cancer of the colon was

TABLE III.—*The Inter-relation of Some Cancers*

| | Correlation coefficient | Type of data | Note |
|---|---|---|---|
| Colon and stomach | −0·7364 | Mortality rates | Wynder and Shigematsu (1967) |
| | −0·7112 | Incidence rates | Countries considered by Wynder and Shigematsu (1967) |
| | −0·1630 | Incidence rates | Countries considered in dietary analysis |
| | −0·1734 | Incidence rates | Countries listed by Doll (1969) |
| Colon and breast | 0·8104 | Incidence rates | Countries considered in dietary analysis |
| | 0·7999 | Incidence rates | Countries listed by Doll (1969) |

FIG. 1.—Correlation between colon and breast cancer rates.

highly correlated with cancer of the breast (Fig. 1). This relationship was also seen when all the incidence data available were considered (Table III) but neither cancer was significantly correlated with cancer of the stomach (Fig. 2, Table III). However, if the data for countries considered previously were extracted a negative correlation between cancer of the colon and stomach was seen.

The present analysis includes data for underdeveloped countries not considered previously (Table I).

*Relation of cancers to dietary and socio-economic factors*

No significant correlation between the incidence of stomach cancer and any of the nutritional or economic factors considered was detected and these results are not presented in detail. Correlation coefficients for breast and colon cancer incidence with the various factors considered are shown in Fig. 3. Both cancers were highly correlated with indicators of affluence such as a high fat diet rich in animal protein and the availability of motor vehicles but the correlation with fat and animal protein was higher than for the other factors.

The use of multiple regression analysis enabled additional factors such as *per capita* income, motor vehicles and radio receivers per head of population to be considered together with the dietary factors. Such analyses showed that not

FIG. 2.—Correlation between colon and stomach cancer rates.

only were fat and animal protein more strongly correlated than socio-economic variables with the incidence of breast and colon cancer but also that diet provided significant additional information after socio-economic variables had been allowed for. These analyses did not enable us to separate the important dietary factors. An example of a multiple regression analysis is given in Table IV. This demonstrates that the correlation of motor vehicles with cancer of the colon could be explained in terms of the correlation between motor vehicles and " combined fat " but that correlation of " combined fat " with colon cancer could not be explained in terms of the correlation between " combined fat " and motor vehicles. However, these analyses also show that the correlation between " combined fat " and animal protein was too close for them to be distinguished.

TABLE IV.—*Multiple Regression Analyses with Colon Cancer Rate as the Dependent Variable*

| Source of variation | Degrees of freedom | Sum of squares | F |
|---|---|---|---|
| Total | 36 | 2508·67 | |
| Regression on combined fat alone | 1 | 1863·79 | |
| Regression on vehicles alone | 1 | 1506·54 | |
| Regression on vehicles after combined fat | 1 | 67·30 | 3·96 N.S. |
| Regression on combined fat after vehicles | 1 | 424·55 | 24·99 $P < 0{\cdot}01$ |
| Deviation | 34 | 577·58 | |
| Regression on combined fat alone | 1 | 1863·79 | |
| Regression on animal protein alone | 1 | 1857·18 | |
| Regression on animal protein after combined fat | 1 | 53·22 | 3·12 N.S. |
| Regression on combined fat after animal protein | 1 | 59·83 | 3·44 N.S. |
| Deviation | 34 | 591·66 | |

FIG. 3.—Correlation coefficients between cancer rates and dietary and other factors.

### DISCUSSION

### *Cancer of the stomach*

In some countries a high incidence of cancer of the stomach is associated with a low incidence of cancer of the colon. Indeed some investigators have suggested that a negative correlation exists between these cancers. The data examined here include a group of countries not considered by Wynder and Shigematsu (1967) and when these areas (Table I, Fig. 2) are included there is no significant correlation between the rates for the two sites. Similarly the inverse relationship between cancer of the stomach and protein intake (Gregor *et al.*, 1969) is not seen. These findings are not surprising when the existence of several pathological types of stomach cancer is considered.

### *Cancers of the colon and breast*

Cancers of the colon and breast are highly correlated with each other and with a high fat and animal protein diet (Fig. 1 and 3). The analysis confirms the finding of Gregor *et al.* (1969) that cancer of the colon is correlated with an animal protein diet and further substantiates the work of Wynder and Shigematsu (1967) on the importance of fat. The results with fibre were disappointing. No significant correlation between fibre, as defined here, and cancer or any other factor was demonstrated. Before these analyses were performed a negative correlation between fibre and income and fibre and fat was expected. This failure may in part be explained by the nature of the data for fibre intake and in part by the lack of definition as to the nature of fibre. But when this absence of correlation is considered in conjunction with the data on sugar and sweets, it does suggest that the putative role of refined carbohydrate in cancer of the colon (Burkitt, 1971) requires much further investigation before any conclusion can be drawn.

The hypothesis linking the intestinal bacteria with the aetiology of cancers of the colon and the breast requires that these cancers be linked to a high fat diet. We might explain the relation of breast cancer to fat and animal protein in terms of greater oestrogen synthesis both by the body and the intestinal flora when a rich diet is consumed (Hill *et al.*, 1971b). The role of fats and bile salts in the aetiology of cancer of the colon has been discussed previously (Hill *et al.*, 1971a; Drasar and Hill, 1972).

Although these studies require amplification through dietary surveys linked to epidemiological studies they do provide good grounds for the assumption that cancers of the colon and breast are related to dietary factors and within the limits of the available data point to the relative significance of fat and protein.

This paper is part of a larger study on cancer of the colon. We would like to thank the Cancer Research Campaign and the Wellcome Trust for financial support.

### REFERENCES

Aries, V., Crowther, J. S., Drasar, B. S., Hill, M. J. & Williams, R. E. O. (1969) Bacteria and the Etiology of Cancer of the Large Bowel. *Gut*, **10**, 334.

Burkitt, D. P. (1971) Neglected Leads to Cancer Causation. *J. natn. Cancer Inst.*, **47**, 913.

Cruickshank, E. W. H. (1946) *Food and Nutrition: the Physiological Bases of Human Nutrition.* Edinburgh: Livingstone.

Davidson, S. & Passmore, R. (1963) *Human Nutrition and Dietetics.* Edinburgh: Livingstone.

Doll, R. (1969) The Geographical Distribution of Cancer. *Br. J. Cancer*, **23**, 1.

Drasar, B.S. & Hill, M. J. (1972) Intestinal Bacteria and Cancer. *Am. J. clin. Nutr.*, in press.

F.A.O. (1969) *The State of Food and Agriculture.* Rome: Food and Agriculture Organisation.

Gregor, O., Toman, R. & Prasova, F. (1969) Gastrointestinal Cancer and Nutrition. *Gut*, **10**, 1031.

Hill, M. J., Drasar, B. S., Aries, V., Crowther, J. S., Hawksworth, G. & Williams, R. E. O. (1971a) Bacteria and the Aetiology of Cancer of the Large Bowel. *Lancet*, i, 95.

Hill, M. J., Goddard, P. & Williams, R. E. O. (1971b) Gut Bacteria and the Aetiology of Cancer of the Breast. *Lancet*, ii, 472.

United Nations Organisation Statistical Yearbook (1970). New York: United Nations.

Walker, A. R. P. (1971) Diet, Bowel Motility, Faeces Composition and Colonic Cancer. *S. Afr. med. J.*, **45**, 377.

Wynder, E. L. & Shigematsu, F. (1967) Environmental Factors of Cancer of the Colon and the Rectum. *Cancer, N.Y.*, **20**, 1520.